# The Effects of Isotretinoin on The Menstrual Cycle: A Cross-Sectional Study

**DOI:** 10.3390/clinpract12060095

**Published:** 2022-11-10

**Authors:** Ghadah Alhetheli, Sadin Alhazmi, Shumukh Almutairi, Samar Alharbi, Norah Alharbi, Maha Alsweed, Mohammed Saleh Al-Dhubaibi, Jolan Alsaud, Lina Asiri

**Affiliations:** 1 Division of Dermatology and Cutaneous Surgery, College of Medicine, Qassim University, Buraydah 52571, Saudi Arabia; 2 College of Medicine, Qassim University, Buraydah 52571, Saudi Arabia; 3 Departments of Dermatology, College of Medicine, Shaqra University, Dawadmi 11911, Saudi Arabia

**Keywords:** reproductive health, isotretinoin, menstruation, amenorrhea, menstrual cycle

## Abstract

Menstrual irregularities during isotretinoin therapy, including amenorrhea, can cause a great deal of health-status uncertainty such as the possibility of pregnancy. This study aimed to evaluate the effects of isotretinoin treatment on the menstrual cycle. This cross-sectional study was conducted among females aged between 15–45 years taking isotretinoin for acne. Descriptive statistics were used in the form of frequencies and percentages to represent categorical variables. Pearson’s chi-squared test was performed to assess the relationship between some of the variables with menstrual irregularities. A logistic regression model was performed to assess the risk factors for developing menstrual irregularities during isotretinoin therapy. Of participants with a known regular menstrual cycle, 10.4% were found to have irregularity in their cycle after starting the drug (*p* < 0.001). Amenorrhea was the most commonly reported menstrual irregularity in isotretinoin-treated females. Our results showed that single females, those who took isotretinoin for 10–12 months and who were concurrently taking hormonal contraceptives all have a statistically significant higher risk of developing menstrual irregularities than others. In conclusion, we found that a statistically significant number of participants with a regular menstrual cycle pre-isotretinoin intake developed irregularity in their cycle after starting the drug. The mechanism of how isotretinoin influences female hormonal imbalances, thereby affecting menstrual irregularities is still poorly understood and needs to be clarified in further clinical studies.

## 1. Introduction

Isotretinoin was first approved by the Food and Drug Administration (FDA) in 1982 for the treatment of severe acne, and it has been used ever since [1]. Isotretinoin (13-cis-retinoic acid) is a synthetic vitamin A derivative, and it remains the most effective acne medication due to its sustained long-term effects. Moderate acne that does not respond to conventional therapies or relapses frequently after finishing oral antibiotics, as well as severe acne, both benefit most from systemic isotretinoin therapy [2,3]. Also, isotretinoin is used for many follicular diseases such as rosacea, seborrhoea, acne inversa, and Propionibacterium folliculitis [4,5]. There are a variety of other skin conditions that can benefit from isotretinoin treatment, including granuloma annulare, discoid lupus erythematosus (DLE), Grover disease, transient acantholytic dermatosis (TAD), and extensive actinic keratosis in sarcoidosis [6,7,8,9,10]. In addition, it is utilized to prevent squamous cell carcinoma of the skin (SCC) [11]. It has been used as an adjuvant in neuroblastoma [12]. Isotretinoin is a systemic retinoid “derivative of vitamin A” which is adequately absorbed following oral administration. The recommended dosage range for moderate to severe acne is from 0.5 to 1.0 mg/kg/d [1]. The exact mechanism of action of this drug is not well understood. However, isotretinoin is the only medication that directly affects all of the four primary etiological factors involved in acne including the normalization of follicular desquamation, the decrease in sebaceous gland activity, the suppression of Cutibacterium acne proliferation, and anti-inflammatory effects [12,13,14]. This drug is found to have a strong impact on cell-cycle progression, cellular differentiation, cell survival, and apoptosis [15,16]. By suppressing hyperkeratinization, isotretinoin significantly reduces comedogenesis. At the same time, it does not play a role in influencing the keratinocytes’ metabolic activity, so the precise mechanisms by which it accomplishes this remain unclear [17,18].

Isotretinoin is used to treat severe nodulocystic acne, and many women of reproductive age are exposed to this drug. Studies in different countries around the world have shown that isotretinoin treatment could affect the menstrual cycle [1,19]. Menstrual irregularities during isotretinoin therapy, including amenorrhea, can cause a great deal of concern regarding the possibility of pregnancy or permanent menstrual cycle irregularity. A case reported by Cox NH., of a 14-year-old girl who had not menstruated while undergoing a ten-week course of isotretinoin, describes menstruation resuming ten days after stopping the medication. The normal menstrual pattern in this patient prior to and after treatment with isotretinoin indicated that amenorrhea for two cycles during treatment was a medication-related incident [20]. In a study conducted by Saljoughi N, et al. on 50 patients treated with isotretinoin for acne vulgaris, 34% of treated patients were found to have menstruation-related side effects, such as amenorrhea and dysmenorrhea, and oligomenorrhea [21].

Furthermore, it is stated that many amenorrhea cases are underreported due to the growing use of oral contraceptive pills (OCPs) during isotretinoin therapy [20]. There is no conclusive data about the side effects of isotretinoin on the menstrual cycle. However, some females experience menstrual irregularities during isotretinoin treatment [20,21]. There are no previously reported studies in Saudi Arabia about the side effects of isotretinoin on the menstrual cycle of female patients. Hence, we designed this study to assess the effects of isotretinoin treatment on the menstrual cycle among females between the age of 15 to 45 years, to identify the type of menstrual irregularities, the dose of the isotretinoin associated with these irregularities, and whether certain menstrual irregularities continued despite drug discontinuation.

## 2. Materials and Methods

### 2.1. Study Design and Participants

This questionnaire-based cross-sectional study was conducted from September 2021 to December 2021. The study included all females between 15 and 45 years old who had taken isotretinoin in the Qassim Region of Saudi Arabia. Females below 15 years and above 45 years or who did not complete the questionnaire, and those who did not approve the informed consent were excluded from the study. The study was approved by the Local Ethical Approval Committee at Qassim University (Project number: 19-14-12). All participants provided written informed consent and were able to withdraw from the study at any stage. The data were kept confidential with the primary investigator and only used for the purposes described in the study objectives. The participants were identified by a numerical listing. A validated questionnaire was used to record the participants’ responses [22]. A minimum sample size of 387 was calculated considering the total population of Qassim to be 1.5 million with an expected prevalence (P) of 35% for acne (based on previous studies), q = 1 − P, d = absolute error or precision, which is 5% (0.05), Z = the standard normal variation at 5% type 1 error (*p*-value ˂ 0.05), which is 1.96. A mixture of convenience and snowball sampling was used to achieve the required sample size. We assumed to keep 10–15% more of the calculated minimum sample, considering a margin of error of 2–3%.

The questionnaire was pretested on a sample of 15 participants who fulfilled the inclusion criteria in order to increase the validity of the content of the measuring instrument. In addition, to evaluate the internal consistency of the items in this questionnaire, Cronbach’s alpha was calculated for the overall questionnaire (0.752) as well as for each of the domains on its own from the responses of the participants [22].

The questionnaire was distributed through online platforms and also during female social gatherings. The questionnaire had two language versions (Arabic and English), and participants were required to answer one of them at their convenience. The questionnaire had three sections. In the first part, a statement of anonymity, confidentially, and research purpose was described at the beginning of the survey, where participants were given the freedom to respond or not. The second part had items that recorded the sociodemographic and menstruation details (Age, marital status, age at puberty, menstruation status, number of days between each menstrual cycle, and menstruation duration). The final part of the questionnaire had items related to isotretinoin use and its effects on participants’ menstrual characteristics.

### 2.2. Data Management and Analysis

All of the responses were initially entered into a single Microsoft Excel sheet, and data cleaning was done by one of the investigators. The data were then subjected to statistical analysis by an independent biostatistician. IBM Statistical Package for the Social Sciences version 23.0 (SPSS Inc., Chicago, IL, USA) was used for the analysis. Statistical analysis included mainly descriptive statistics with proportions and percentages for categorical variables and relationships (Pearson’s Chi-squared test) between the categorical variables. A binary logistic regression was used to assess the risk factors considering ‘menstruation after isotretinoin’ as a dependent variable. A *p*-value < 0.05 was considered statistically significant.

## 3. Results

A total of 437 females of reproductive age were included in the study. Sociodemographic data are shown in Table 1. There were 258 participants (59%) between the ages of 21–25 years old. As for marital status, 384 of them (87.9%) were married. There were 107 participants (24.5%) who had reached puberty at the age of 13 years. As for menstrual cycle irregularities before using isotretinoin, 52 participants (11.9%) reported that they had irregular menstruation while 133 participants (30.4%) had a menstrual cycle of more than 28 days, and 53 participants (12.1%) had a menstruation duration of more than seven days (Table 1).

When we assessed the isotretinoin-related practices, 200 participants (45.8%) reported that they started using isotretinoin at the age of 21–25 years, 149 participants (34.1%) used a dosage of 40 mg daily (0.5 mg/kg/day), 45 participants (10.3%) had a treatment course for 10–12 months. As for menstrual cycle irregularities after starting isotretinoin, it was reported by 83 females (19.0%) that their menstrual cycle was ‘not regular’ after using isotretinoin, where 60 participants (72.3%) had periods once every two months, while 2 participants (2.4%) had a period once in a year, and 13 participants (15.7%) had periods once in 3 months. Also, three participants (3.6%) had a period once in six months, two participants (2.4%) had a period once in 9 months, and three participants (3.6%) had no menstrual cycle after starting isotretinoin. As for menstruation changes after starting isotretinoin, it was reported by 175 participants (40.0%) that they did not experience any change in their menstrual cycle after using isotretinoin, while 123 participants (28.1%) experienced some changes in their menstruation. These changes were filed in three categories: prolonged menstruation in 29 participants (6.6%), shortened menstruation in 29 participants (6.6%), and late period in 65 participants (14.9%). Of note, 139 participants (31.8%) did not respond regarding changes to their menstruation after using isotretinoin. It was reported by 15 participants (3.4%) that they had taken some hormonal therapy along with isotretinoin, and 60 participants (13.7%) had taken hormonal contraceptives along with isotretinoin (Table 2).

It was found that 52 participants (11.9%) had before using isotretinoin, and 83 participants (19%) had irregular menstrual cycles after using it. When we assessed the relationship between menstruation before and after using isotretinoin, we found that 40 participants (10.4%) from those with known regular periods before isotretinoin treatment had reported irregular periods after using isotretinoin, which is statistically highly significant (*p* < 0.001). There was no significant association observed in menstruation irregularities after using isotretinoin with the age of the females (*p* = 0.075). Single females reported significantly higher menstruation irregularities after using isotretinoin (*p* = 0.008). It was found that there was no statistically significant association between those who had delayed menarche, 142 participants (32.5%), and menstrual irregularities after starting the drug (*p* = 0.190). It was found that females who had prolonged menstrual cycles (>28 days) reported significantly more irregularities in menstruation after using isotretinoin (*p* < 0.001). There were no statistically significant associations observed for menstruation irregularities after using isotretinoin with age at which isotretinoin use started (*p* = 0.107) or with hormonal treatment while using isotretinoin (*p* = 0.919). However, we observed that females who had isotretinoin treatment for more than 10 months and those who had concurrently taken hormonal contraceptives reported significantly higher menstrual irregularities after taking isotretinoin (*p* < 0.05) (Table 2 and Table 3).

A logistic regression model analysis showed that being single (OR = 4.88 (1.30–18.38), *p* = 0.019), using isotretinoin for more than 10 months (OR = 2.11 (1.02–4.39), *p* = 0.045), and taking hormonal contraceptives (OR = 3.40 (1.66–6.98), *p* < 0.001) were found to be independent risk factors for menstrual irregularities after using isotretinoin (Table 4).

## 4. Discussion

Studies that evaluated the effect of isotretinoin on the menstrual characteristics of women of reproductive age are limited. Young women are more likely to experience menstrual irregularities due to physiologic hormone changes [23]. However, our statistically significant findings showed that 40 participants with known regular menstrual cycles before the commencement of isotretinoin were found to have irregularity in their cycle after starting the drug. This points to the influence of this drug on hormonal changes in females. Evidence shows that isotretinoin, due to its derivation from vitamin A, is highly teratogenic and is therefore strictly contraindicated in women of reproductive age unless effective contraception is taken, often in the form of oral contraceptive pills [24,25]. A study done by Peck et al. reported that in patients treated with isotretinoin, no changes in hormone levels were seen, suggesting a different cause for menstruation irregularities [26]. In our study, amenorrhea was the most commonly reported menstrual irregularity in isotretinoin-treated females, which is similar to the findings of a study done by Christmas [27]. This is supported by the findings of a study done by Lithgow DM and Poliztzer WM, which showed that vitamin A was effective in treating menorrhagia [28]. From the aforementioned, the relationship between delayed menstrual bleeding and the use of isotretinoin in our patients appears to be a paradox for which no concise answer can be established.

Another observation we would like to highlight is the possible impact of stress on the hormonal imbalance in women. It is plausible that severe acne contributes to the development of these menstrual abnormalities. In this context and according to research evidence, females who suffer from severe acne are more likely to become depressed and may experience more menstrual irregularities than those who are not under stress [29,30]. It is true that both females who use isotretinoin and those who do not have the treatment are affected. It is possible that females who become so depressed may lose their confidence which could have a negative impact on their social life. When acne goes away, depression and negative thoughts also tend to disappear [31]. Furthermore, our study findings showed that being a single female, prolonged use of isotretinoin and concurrent use of hormonal contraceptives all increase the risk of developing menstrual irregularities. This is supported by other studies which showed that single females tend to have a double risk of experiencing menstrual irregularities [32]. Therefore, it can be assumed that females who are on isotretinoin may be at increased risk of developing these menstrual irregularities. Being married and having a healthy lifestyle may positively impact the occurrence of menstrual abnormalities, although the reasons for this are not yet clear [33]. Studies showed that menstrual bleeding patterns have been altered by many hormonal contraceptive methods [34,35]. We also observed that females who were on isotretinoin for more than ten months had reported comparatively higher menstrual irregularities. It was reported that isotretinoin therapy for at least four months in a dosage range of 0.7–1.2 mg/kg/day caused menstrual irregularities in 10 of the 40 patients. Interestingly, the menstrual cycles in these affected females returned to normal after the discontinuation of the treatment [36].

Menstruation is contingent upon the balance and proper blood levels of a variety of hormones. It has been claimed that retinoids play a critical role in endometrial development and secretory differentiation, and it was found that women with menorrhagia have lower serum vitamin A levels than healthy individuals [28]. This is supported by the findings of two other studies, which reported that 33.3% and 28.3% of the females had experienced menstrual irregularities undergoing isotretinoin treatment, respectively [19,37]. Furthermore, a study carried out by Saljoughi, N et al. reported that oligomenorrhea, amenorrhea, and dysmenorrhea were seen among 6%, 4%, and 10% of the females who were on an isotretinoin regime, respectively [21]. Therefore, it could be assumed that isotretinoin’s adverse effects are self-limiting, controllable, and dose-dependent, although doctors should closely follow patients due to the risk of more serious complications. The current study findings also showed that females with a prolonged menstrual cycle (>28 days) were found to be more prone to irregular menstrual cycles during isotretinoin treatment. These findings are similar to the findings of a recent study carried out by Akpolat [38]. It is essential to have new guidelines to compensate for these possible adverse effects, as some studies had investigated measurements to avoid other adverse effects such as isotretinoin-associated ocular surface disease and teratogenicity [39,40].

Our research encountered some limitations. Firstly, we used a self-reported questionnaire to record the responses, and this might have called upon recall bias and social desirability. Secondly, there could have been confounding factors when trying to establish a relationship between many variables and menstrual irregularities, as we might not have evaluated many biological, environmental, and psychosocial factors that could affect menstrual characteristics. Thirdly, we did not assess the severity of acne experienced by the participants. Therefore, the authors of this research suggest including more variables when planning future research, and this should preferably follow a prospective design with a larger sample from different geographical locations.

## 5. Conclusions

In conclusion, menstrual irregularities during isotretinoin treatment constitute a state of uncertainty that can cause anxiety. We clearly found that being a single female, taking isotretinoin for a prolonged time of more than 10–12 months, and the concurrent taking of hormonal contraceptives all showed a higher risk of developing menstrual irregularities. Further, we found that a statistically significant number of participants with regular menstrual cycles pre-isotretinoin intake had irregularity in their cycle after starting the drug. It is unclear what causes menstrual irregularities during isotretinoin therapy and the scientific interpretations behind this wide range of irregular menstrual patterns. Thus, it is essential to have new guidelines to compensate for these possible adverse effects. Interpretation of menstrual irregularities associated with isotretinoin administration needs to be elucidated further in larger clinical prospective studies.

## Figures and Tables

**Table 1:** Baseline characteristics.

		Frequency	Percent
Age (years)	15–20	97	22.2
	21–25	258	59.0
	26–30	66	15.1
	31–35	10	2.3
	36–40	5	1.1
	41–45	1	0.2
Marital status	Married	53	12.1
	Single	384	87.9
Age at puberty (years)	10	26	5.9
	11	54	12.4
	12	108	24.7
	13	107	24.5
	14	89	20.4
	15	29	6.6
	16	11	2.5
	>16	13	3.0
Menstruation	Not regular	52	11.9
	Regular	385	88.1
Number of days between each menstrual cycle	<28 days	155	35.5
	28 days	149	34.1
	>28 days	133	30.4
Menstruation duration	1 day	2	0.5
	1–3 days	19	4.3
	5 days	123	28.1
	7 days	240	54.9
	>7 days	53	12.1

**Table 2:** Isotretinoin-related practices and menstrual characteristics.

		Frequency	Percent
Age at which started using isotretinoin (years)	15–20	198	45.3
	21–25	200	45.8
	26–30	27	6.2
	31–35	6	1.4
	36–40	4	0.9
	41–45	2	0.5
Dosage of isotretinoin used	10 gm	66	15.1
	20 gm	139	31.8
	30 gm	83	19.0
	40 gm	149	34.1
Treatment duration of isotretinoin (months)	1–3 months	93	21.3
	4–6 months	196	44.9
	7–9 months	103	23.6
	10–12 months	45	10.3
Menstrual cycle after using isotretinoin	Not regular	83	19.0
	Regular	354	81.0
If not regular (*n* = 83)	Has not come since using isotretinoin	3	3.6
	Once a year	2	2.4
	Once every 3 months	13	15.7
	Once every 6 months	3	3.6
	Once every 9 months	2	2.4
	Once every two months	60	72.3
Menstruation changes after using isotretinoin	No change	175	40.0
	Menstruation prolonged	29	6.6
	Menstruation shortened	29	6.6
	Period late	65	14.9
	Not respond to the question	139	31.8
Taking hormonal treatment while using isotretinoin	No	422	96.6
	Yes	15	3.4
Taking hormonal contraceptives	No	377	86.3
	Yes	60	13.7
Regular period before using hormonal contraceptives	No	70	16.0
	Yes	367	84.0

**Table 3:** Menstrual cycle and mensuration characteristics before and after isotretinoin.

		Menstruation after Using Isotretinoin		*p*-Value *
		Regular	Not Regular	
Menstruation before isotretinoin	Regular	345 (89.6%)	40 (10.4%)	<0.001 *
	Not Regular	9 (17.3%)	43 (82.7%)	
Age	15–20	70 (72.2%)	27 (27.8%)	0.075
	21–25	211 (81.8%)	47 (18.2%)	
	26–30	57 (86.4%)	9 (13.6%)	
	31–35	10 (100.0%)	0 (0.0%)	
	36–40	5 (100.0%)	0 (0.0%)	
	41–45	1 (100.0%)	0 (0.0%)	
Social status	Married	50 (94.3%)	3 (5.7%)	0.008 **
	Single	304 (79.2%)	80 (20.8%)	
Puberty	Normal	244 (82.7%)	51 (17.3%)	0.190
	Delayed	110 (77.5%)	32 (22.5%)	
Number of days between each menstrual cycle	28 days	136 (91.3%)	13 (8.7%)	<0.001 *
	<28 days	137 (88.4%)	18 (11.6%)	
	>28 days	81 (60.9%)	52 (39.1%)	
Menstruation duration	≤7 days	312 (81.3%)	72 (18.8%)	0.727
	>7 days	42 (79.2%)	11 (20.8%)	
Age at which started using isotretinoin	15–20	155 (78.3%)	43 (21.7%)	0.107
	21–25	165 (82.5%)	35 (17.5%)	
	26–30	26 (96.3%)	1 (3.7%)	
	31–35	5 (83.3%)	1 (16.7%)	
	36–40	2 (50.0%)	2 (50.0%)	
	41–45	1 (50.0%)	1 (50.0%)	
Dosage of isotretinoin used	10 gm	49 (74.2%)	17 (25.8%)	0.450
	20 gm	113 (81.3%)	26 (18.7%)	
	30 gm	70 (84.3%)	13 (15.7%)	
	40 gm	122 (81.9%)	27 (18.1%)	
Treatment duration for isotretinoin	≤9 months	323 (82.4%)	69 (17.6%)	0.029
	≥10 months	31 (68.9%)	14 (31.1%)	
Taking hormonal treatment while using isotretinoin	No	342 (81.0%)	80 (19.0%)	0.919
	Yes	12 (80.0%)	3 (20.0%)	
Taking hormonal contraceptives	No	312 (82.8%)	65 (17.2%)	0.019 **
	Yes	42 (70.0%)	18 (30.0%)	
Regular periods before using hormonal contraceptives	Regular	313 (85.3%)	54 (14.7%)	<0.001 *
	Not regular	41 (58.6%)	29 (41.4%)	

*: highly significant differences (*p* ≤ 0.001), **: mildly significant difference (*p* < 0.05).

**Table 4:** Logistic regression.

		Odds Ratio (OR)	95% C.I. for OR		*p*-Value
			Lower	Upper	
Variables	Age > 25 years	0.61	0.24	1.52	0.285
	Marital status = Single	4.88	1.30	18.38	0.019 **
	Delayed puberty	1.52	0.90	2.58	0.122
	Age started using isotretinoin ≥ 25 years	1.17	0.34	4.03	0.801
	Duration of using isotretinoin > 10 months	2.11	1.02	4.39	0.045 **
	Dose of isotretinoin = 40 mg	0.98	0.57	1.69	0.936
	Taken hormonal contraceptive	3.40	1.66	6.98	0.001 *
	Taken other hormonal treatments	0.95	0.20	4.58	0.950
	Constant	0.03			0.000

OR: odds ratio, C.I.: confidence interval *: highly significant differences (*p* ≤ 0.001), **: mildly significant difference (*p* < 0.05).

## Data Availability

Not applicable.
